# The Modified Vaccination Technique

**DOI:** 10.3390/vaccines7010001

**Published:** 2018-12-21

**Authors:** Arpad Barabas, Chad Cole, Zoltan Kovacs, Erno Kovacs, Rene Lafreniere

**Affiliations:** 1 Department of Surgery, University of Calgary, Calgary, AB T2N 1N4, Canada; kovacsz@ucalgary.ca (Z.K.); rlafreni@ucalgary.ca (R.L.); 2 Department of Neurosurgery, University of Utah, Salt Lake City, Utah, UT 84112, USA; Cole.nsgy@gmail.com; 3 Department of Medical Genetics, University of Calgary, Calgary, AB T2N 1N4, Canada; erno.kovacs@ucalgary.ca

**Keywords:** Heymann nephritis, antibody information transfer, immune complex

## Abstract

In addition to active and passive immunizations, there is a third method of immunization, the modified vaccination technique, which is based on injecting a combination of target antigens and antibodies against this antigen. The vaccine is essentially comprised of immune complexes with pre-determined immune-inducing components. When such an immune complex (target antigen × antibody against the target antigen) with a slight antigen excess is administered, it evokes a corrective immune response by the production of the same antibody with the same specificity against the target antigen that is present in the immune complex (pre-determined immune response).

## 1. Introduction

The discovery and implementation of an active immunization technique, using cow pox by Edward Jenner (1749–1823), was the most significant first step in the prevention and spread of small pox, a highly contagious and infectious disease of humans. In 1891, Emil von Behring introduced passive immunization for the protection of diphtheria and, later on, against tetanus. Since then, scientists have developed new vaccines (using various techniques to attenuate, inactivate, and genetically engineer vaccines) to protect humankind by mass immunization programs.

**Figure 1:**
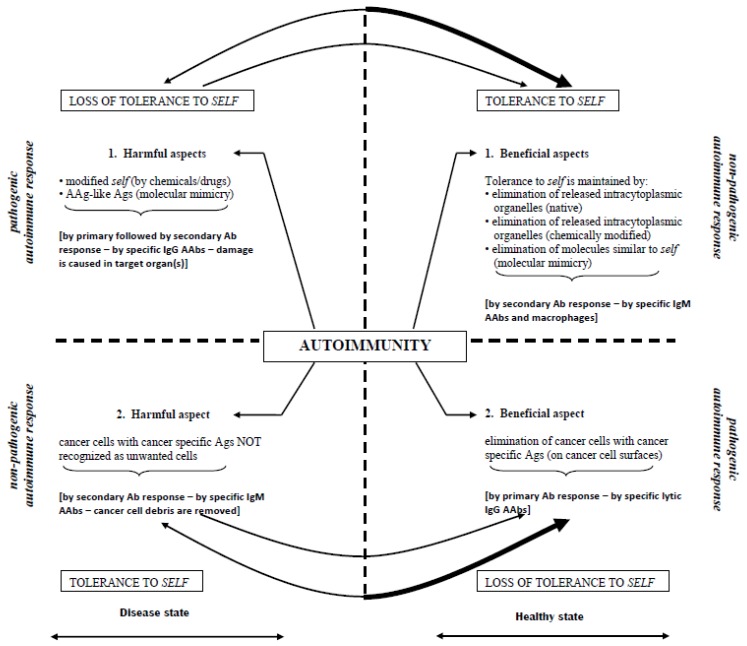
Induction of autoimmunity represented by beneficial and harmful immune responses against self. Regaining tolerance to self in both autoimmune diseases and cancer by the removal of intracytoplasmic components and elimination of cancer cells with cancer-specific antigens, respectively. (Figure reproduced by permission from *BioProcessing Journal*, 2007 Winter, 6(4), 12–18). Abbreviations: AAg, autoantigen; AAbs, autoantibodies; Ab, antibody; Ag, antigen; IgG, immunoglobulin G; IgM, immunoglobulin M; MVT, modified vaccination technique.

Our inability to deal with increasing amounts of pathogens at the present time is due in part to their ability to mutate, but also due to our inability to find new drugs and vaccination protocols. To do so, we must first understand the mechanisms of the immune system. Figure 1 depicts two beneficial and two harmful immune responses against self. In an autoimmune disease, the target is to remove intracytoplasmic components, and in cancer, it is to eliminate cancer cells with cancer-specific antigens (ags).

Currently, there are two vaccination protocols: active and passive immunization [1,2,3,4,5,6]. Both have had limited success in correcting endogenous ag-caused afflictions, i.e., autoimmune diseases and cancer [2,4,7,8,9,10,11,12,13,14,15,16,17,18]. Based not only on their own observations but also on the extensive research work of others [19,20,21,22,23,24,25,26,27,28,29,30,31,32,33,34,35,36,37,38], Barabas and colleagues have developed a third vaccination technique (which is both prophylactic and therapeutic) that promises to effectively deal with these afflictions; they call this the modified vaccination technique (MVT) [39,40,41,42,43,44,45,46,47].

## 2. Modified Vaccination Technique (MVT)

The MVT, developed in the Barabas laboratory for the prevention and/or treatment of disorders that are difficult or impossible to treat (including cancer and autoimmune diseases), was employed in several experiments to investigate its utility [39,40,41,42,43,44,45,46,47].

**Figure 2:**
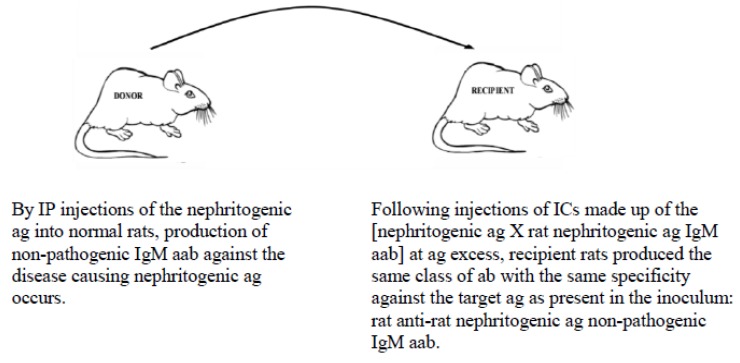
AIT by initiating/maintaining non-pathogenic IgM ab response against the autoimmune disorder causing nephritogenic aags for the termination of the autoimmune disease. The increased levels of rat nephritogenic IgM aabs neutralize both circulating disease maintaining modified ag and disease contributing native aag. Disease process is terminated and tolerance to self is re-established. Abbreviations: aab, autoantibody; aag, autoantigen; AIT, antibody information transfer; ab, antibody; ag, antigen; IC, immune complex; IgM, immunoglobulin M; IP, intraperitoneal.

In an experimental autoimmune kidney disease model, slowly progressive Heymann nephritis (SPHN; somewhat similar to Lupus) was induced in rats by the injection of rat kidney fraction 3 (rKF3) incorporated in Alum or in an azo rKF3 ag. Once the disease was in its progressive phase, rats were injected with an immune complex (IC): (rKF3 ag × rat anti-rat rFK3 ag non-pathogenic immunoglobulin M (IgM) antibody (ab)). The non-pathogenic IgM abs were responsible for the downregulation/termination of the autoimmune kidney disease by assisting in the removal of the nephritogenic ags from the system. As seen in Figure 2, several of our experiments have proven the prevention/termination of SPHN by the injection of the IC, which not only represents the MVT, but also provides a predetermined immune response outcome with the same specificity against the target ag (rKF3) that is present in the inoculum [39,40,41].

**Figure 3:**
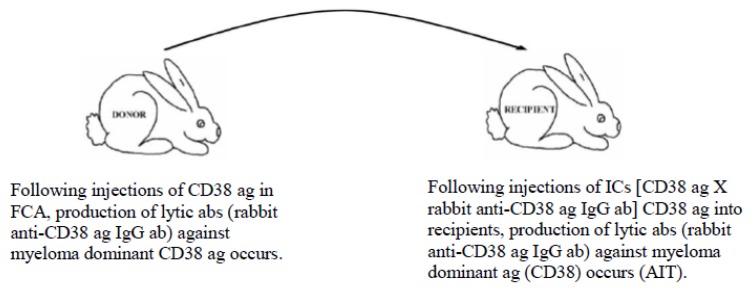
AIT by initiating/maintaining lytic ab response against CD38 cancer specific ag. Recipient rabbits produced the same class of ab with the same specificity against the target ag as present in the inoculum, namely rabbit anti-CD38 ag lytic IgG abs that was able to lyse (eliminate) cancer cells in vitro and in vivo in a complement fixation test. Abbreviations: ab, antibody; ag, antigen; AIT, antibody information transfer; CD38, cancer specific antigen; FCA, Freund’s complete adjuvant; IgG, immunoglobulin G.

In cancer experiments, we produced lytic immunoglobulin G (IgG) (which is cancer-cell killing) abs (detected with a complement fixation test) against cancer specific ags in donor rabbits injected with CD38 (cluster of differentiation 38) ag in Freund’s complete adjuvant [47]. Using the MVT, we achieved antibody information transfer (AIT) using ICs in recipient rabbits (CD38 ag × rabbit anti-CD38 ag lytic IgG ab). Following the administration of ICs, recipient animals produced the same ab with the same specificity against the target ag (i.e., cancer-specific CD38 ag) present in the IC, as seen in Figure 3. Repeated injections of the IC maintained high-titre lytic IgG abs against the cancer specific CD38 ag, resulting in cancer cell death/elimination by developing pathogenic IgG autoantibodies (aabs) [47].

The next step for the MVT should be to investigate its effect in patients with cancer in a well-planned clinical trial. The ingredients (i.e., the soluble small molecular weight cancer poly-specific target ags and poly-specific pathogenic lytic IgG abs targeting ags associated with the target cancer) are available for some cancers. Unlike conventional vaccines that contain an ag (and adjuvants) to induce active immunity, or passive immunization with hyper-immune sera or gamma-globulin preparation (a laboratory-made preparation for injection against a well-defined ag), the modified vaccine is composed of components present in both active and passive immunizations, i.e., ags and specific abs against the target ag. The modified vaccine is comprised of an exogenous or endogenous ag and specific abs against target ags in the form of an IC. Such ICs can evoke predetermined immune responses in recipients by producing the same class of immunoglobulins with the same specificity against the target ags that are present in the ICs.

We believe that the MVT is a highly effective immunization method to manage exogenous (acute/chronic infectious) and endogenous ag initiated and maintained disorders, such as autoimmune diseases and cancer [48]. In our view, this new vaccination technique is the missing link for preventing/treating acute/chronic disorders specifically and with minimal side effects, using the immune system’s natural abilities to respond to corrective information.

## 3. Antibody Information Transfer (AIT)

The presentation of an ag to cells of the immune system determines the immune response outcome. The information-based immune system will respond to exogenous and endogenous ags. Most often, the response is beneficial, but at times harmful, causing disease (by inciting agents like cigarette smoke, radiation, certain drugs, chemicals, infectious agents, etc.) [49,50,51,52,53,54,55,56,57,58,59,60,61,62,63,64,65].

AIT can be initiated when prepared ICs made up of target ags and abs against the target ags are prepared for administration for prophylactic and therapeutic use to evoke beneficial immune responses. However, ICs can evoke harmful immune responses as well, e.g., when they initiate pathogenic IgG ab responses against target ags, as in autoimmune diseases.

In many ways, AIT is similar to immune events that occur during secondary ab response where ICs are processed by dendritic cells and other immunologic cell types and pass necessary information/instruction to plasma cells to produce/replicate immunoglobulins with the same structure/function present in the IC. AIT can down-regulate pathogenic immune responses driven by pathogenic IgG ab-mediated events, as in the experimental autoimmune kidney disease SPHN, by injections of ICs comprised of (nephritogenic ag × rat anti-rat nephritogenic ag non-pathogenic IgM aab) [39]. However, it can also up-regulate pathogenic IgG ab-mediated events against a target ag—present on the outer surfaces of cancer cells (so-called cancer-specific ags)—by specific lytic IgG abs, such as (cancer-specific ag × homologous anti-homologous cancer-specific ag pathogenic lytic IgG aabs) [47]. Other research scientists have used ICs for treatment with limited success [66,67,68,69,70].

The MVT promises to prevent and with equal effectiveness treat/terminate chronic ailments (such as cancer, autoimmune diseases, acute and chronic infections) by re-directing the immune system’s function specifically (both prophylactically and therapeutically) to achieve (by injections of suitably prepared ICs) [39,40,41,42,43,44,45,46,47,48,71,72,73]:

- Down-regulation of pathogenic IgG aab responses in autoimmune diseases, thereby preventing/terminating immune events that attempt to destroy self targets (Figure 2);
- Up-regulation of pathogenic immune responses against cancer-specific ags to lyse cells (irrespective of their locations (metastatic spread)) (Figure 3); and
- Initiation of immune responses against agents causing acute/chronic infections (e.g., malaria, Ebola, cholera, influenza, etc.).

## 4. Therapeutic Immunization Program

It is evident that modifying agents such as sunshine, drugs, chemicals, radiation, implants, adjuvants, fumes, bacterial infections, etc., can alter autoantigens (aags) and that these altered aags may cause autoimmune diseases [49,50,51,52,53,54,55,56,57,58,59,60,61,62,63,64,65].

In order to cause SPHN, a modified self ag has to be presented to cells of the immune system, for example, a haptenized nephritogenic ag [51]. Such haptenized nephritogenic ag is only slightly different from normal self ag, i.e., nephritogenic ag, albeit different enough to cause a pathogenic IgG aab response able to target the brush border-located nephritogenic ag of renal proximal convoluted tubules. How can we terminate the ongoing damage to the nephritogenic ag? Fortunately, this can be done by appropriate application of the MVT [41].

The MVT can remove from the circulation both native and modified ags, i.e., haptenized nephritogenic ags. A lack of pathogenic IgG aab-producing ags in the circulation will result in a rapid decrease/termination of disease-causing cross-reactive aabs (rat anti-rat nephritogenic ag IgG ab).

For preparation of the MVT, nephritogenic ag and rat anti-nephritogenic ag IgM ab are needed. The vaccine is comprised of an IC (nephritogenic ag × rat anti-rat nephritogenic ag IgM ab). Repeated injections of IC at weekly intervals will increase production of the same ab with the same specificity against the target ag (nephritogenic ag) present in the inoculum (AIT). Furthermore, since the IgM ab is cross-reactive, it will also react with the haptenized nephritogenic ag (that initiated/maintained the autoimmune kidney disease, i.e., the immune complex glomerulonephritis). Furthermore, the self ag that contributed to the disease could also contribute to its termination when presented to the immune system by the MVT [73].

## 5. Preventative and Therapeutic Possibilities in Acute and Chronic Immunological Disorders

### 5.1. Example of Vaccination against A Hyper-Acute Infection by the MVT

Hyper-acute infections, such as Ebola, frequently cause death. In brief, suggested vaccination programs with an active immune-inducing program are as follows:

(1) Prophylactic Immunization Program

Need:

- Ebola virus ag (inactivated but immunogenic), laboratory-produced/tested;
- Human anti-Ebola virus ag poly-specific IgG abs, obtained from sera of recovered patients;
- To employ MVT, produce ICs comprised of human Ebola ag × human anti-Ebola neutralizing IgG abs.

The prepared IC can be injected into recipients as is, or in an appropriate adjuvant (used in humans).

(2) Therapeutic Immunization Program

Caregivers, nurses, and occasionally patients may acquire Ebola in epidemic zones of Africa.

Need:

- The same components as above and the same MVT to induce, by repeated injections, high ab titres of human anti-Ebola neutralizing IgG abs. If the modified vaccine is given in time, patients should survive.

To obtain pure components, ICs are made at the point of equivalence and centrifuged and the precipitate is repeatedly washed in saline to obtain, in the IC, pure abs against Ebola-specific ags and pure Ebola-specific ags, i.e., a laboratory-prepared immunogenic but not disease-causing Ebola virus ag. All irrelevant ags are washed away.

### 5.2. Example of Vaccination against an Autoimmune Disease-Causing Ag Using the MVT

There is a steady increase in autoimmune diseases in the world [74]. Furthermore, approximately 8% of the United States population has an autoimmune disease [75]. At present, patients are treated with immunosuppressive agents. These are non-specific, result in no recovery, and often cause side effects [2,4,7,8,9,10,11,12,13,14,15,16,17,18]. However, the Barabas group has described a new vaccination technique that is able to downregulate/terminate an autoimmune kidney disease in rats, which is morphologically and functionally similar to lupus. The MVT was implemented in SPHN during the chronic progressive phase of the disease. SPHN in rats was also treated by the MVT from induction of the disease, resulting in no disease or only very minor evidence of it [39,40,41,42,71,72]. Since preventative steps in humans are usually not pursued or not known, only treatment by MVT will be described.

### 5.3. Example of Vaccination against Cancer-Causing Ags Utilizing the MVT

Approximately one of every three or four people, if they live long enough, will acquire cancer. Most cancers are treated with surgery, chemotherapy, radiation, etc. However, treatments are not specific, have many side effects, and often lead to no recovery. Monoclonal abs targeting the cancer specific ags on the outer aspect of cancer cells have been used. Unfortunately, they are not consistently effective in lysing cancer cells and can have side effects. The most recent approach isolates chimeric antigen receptor (CAR)-T cells from patients, which are engineered to have specificity for the cancer antigen and then adoptively transferred back to the patients. However, this is expensive and only partially effective, with many undesirable side effects.

The MVT is an active immunization program. Relatively small amounts (micrograms) of ags in a small volume of ab solution are injected into recipients in the form of IC. This should induce, by AIT, the development of the same ab with the same specificity that is present in the IC, i.e., cancer cell-killing lytic IgG abs. The vaccine would be comprised of low MW cancer poly-specific ags and homologous anti-cancer poly-specific ag lytic IgG abs. Ideally, in patients with cancer, it should be possible to use appropriate ICs for prophylactic and therapeutic applications to upregulate immune responses to the target aags that initiated and/or propagated the disease.

## 6. Conclusions

The MVT works by AIT; the vaccine—the IC—stimulates production of the same class of immunoglobulin with the same specificity against the target ag that resides in the IC. By AIT, a predetermined ab response is initiated and, by repeated injections of the IC, a predetermined ab response is maintained. We call our vaccination technique MVT because several highly specific immunogenic components have to be prepared, including the final product: the ag and ab against the ag. The combination of these two provides the IC capable of evoking corrective immune responses. Hence, conditions such as autoimmune diseases and cancer in patients may revert back to a normal state of health following treatment with the MVT, without the use of pharmaceuticals.

It is well established that a target ag-evoked specific immune response may correct mishaps that occur in autoimmune diseases and cancer [3]. However, it was not known until recently how to achieve such a desired immune response specifically and without side effects using appropriate self-like and fabricated ags. Concerns about the induction of accelerated tissue-injurious complications (side effects) and the accelerated growth and spread of cancer cells also hindered progress, as did research into T cells (CAR-T cells) with cancer cell-killing properties [76].

The MVT provides the following advantages:

- It is both prophylactic and therapeutic with equal effectiveness (no other vaccination program offers this);
- It can be used against exogenous and endogenous ags (e.g., with the elimination of modified self ag from the system that could otherwise induce an autoimmune disease, etc.);
- It can downregulate/terminate pathogenic immune responses in certain autoimmune diseases by non-pathogenic poly-specific IgM aabs targeting native and modified disease-causing ags;
- It can upregulate pathogenic immune responses against target ags (i.e., cancer-associated and cancer-specific ags) by inducing production of lytic poly-specific IgG aabs;
- It can combine the study of oncology and autoimmunology, making it possible to investigate, from a common perspective, the etiology, pathogenesis, and treatment of two dreaded disorders: cancer and autoimmune disease [77,78];
- When specific ICs are prepared for immunization, only small amounts (micro-gram doses) of ag and abs against the ag are required to evoke the desired immune responses;
- The ICs do not require adjuvants for adequate responses in recipients, although acceptable adjuvants included in the IC will enhance immune response, such as that initiated by the elimination of modified self ags that are causing autoimmune diseases, and enhance ab response to target cancer-specific ags to lyse cancer cells;
- It can be produced cost-effectively and should considerably reduce health care expenses while allowing highly effective treatments to be initiated with minimal delay.
